# In eHealth in India today, the nature of work, the challenges and the finances: an interview-based study

**DOI:** 10.1186/1472-6947-14-1

**Published:** 2014-01-06

**Authors:** Szymon Jarosławski, Gayatri Saberwal

**Affiliations:** 1 Institute of Bioinformatics and Applied Biotechnology, Biotech Park, Electronics City Phase I, Bangalore, 560 100, India

**Keywords:** eHealth, Telemedicine, mHealth, Rural healthcare, Diagnostics, Distant medical education

## Abstract

**Background:**

India is a country with vast unmet medical needs. eHealth has the potential to improve the quality of health care and reach the unreached. We have sought to understand the kinds of eHealth programmes being offered in India today, the challenges they face and the nature of their financing.

**Methods:**

We have adopted an interview-based methodology. The 30 interviews represent 28 organizations, and include designers, implementers, evaluators and technology providers for eHealth programmes.

**Results:**

A range of programmes is being run, including point-of-care in rural and urban areas, treatment compliance, data collection and disease surveillance, and distant medical education. Most programmes provide point-of-care to patients or other beneficiaries in rural areas. Technology is not a limiting factor but the unavailability of suitable health personnel is a major challenge, especially in rural areas. We have identified a few factors that help this situation. Financial sustainability is also a concern for most programmes, which have rarely been scaled up. There are recent for-profit efforts in urban areas, but no reliable business model has been identified yet. Government facilities have not been very effective in eHealth on their own, but collaborations between the government and non-profit (in particular) and for-profit organisations have led to impactful programmes.

**Conclusions:**

It is unlikely that eHealth will have widespread and sustainable impact without government involvement, especially in rural areas. Nevertheless, programmes run solely by the government are unlikely to be the most effective.

## Background

India is a vast country with complex socio-economic characteristics that are reflected in its medical systems. These include an insufficient number of primary care doctors practising in rural and semi-urban areas [1] and an ongoing need to update the knowledge of those who do work in rural areas [2]. Qualified doctors’ practice can diverge widely from standards of care, with many medical practitioners lacking formal qualifications altogether [3]. About 80% of the population depends on non-allopathic medicine [4] and a study of the Indian pharma industry has estimated that the penetration of modern medicine in the country is only 30% [5]. Also, out-of-pocket expenditure, which constitutes around 80% of the total healthcare spending in India [6,7] may be further inflated by travel costs in both urban and rural areas [8]. Thus, many conditions remain untreated or are managed with prescription medicines purchased over-the-counter [8] or by faith healers [9]. Consequently, the 70% of the population that lives in rural areas, in particular, has limited access to adequate health care. Finally, epidemiological data is often unavailable or non-reliable [10], hampering the informed design of preventive health programmes.

The use of Information and Communication Technologies (ICT) for health (eHealth) has the potential to improve all these areas, that is facilitate access to quality health care and to health information as well as improve the quality of health-related data, as has been demonstrated in other developing countries [11] and references therein, [12]. The eHealth based delivery of health-related services can involve remote clinical participation, that may involve mobile communications technology, a sub-category known as mHealth [13].

We have identified numerous reports on eHealth in India. However most of the papers (i) were written by persons involved in running such programmes [14-18]; (ii) reported studies on user attitudes towards eHealth [19-21] or (iii) were literature reviews [16,22,23]. We believed that unlike other methodologies such as case studies of a few programmes, for instance, an interview-based study would give us insights into the experiences and opinions of a range of designers, implementers, evaluators and technology providers, to understand the types of programmes being run, the challenges in running them and the patterns of funding, with implications for sustainability and scale up. Therefore we undertook an interview-based study to understand the kinds of eHealth programmes being offered in India today – the nature of the work, its financing, and the challenges in running the programmes.

The authors have no prior or current links with eHealth programmes, in India or abroad, and therefore have no conflict of interest. They had no preconceived notions about the expected or preferred outcomes of the study and therefore believe that they have had no bias in the formulation of the research question, in data collection or in its interpretation.

## Methods

### Design

We adopted an exploratory, interview-based methodology. Approval for this research was obtained from the Ethics Committee of IBAB and the research was carried out in compliance with the Helsinki Declaration. Prior to conducting the interviews, a detailed questionnaire was constructed based on a review of the peer-reviewed and grey literature about telemedicine in India including publications of the World Health Organization, the mHealth Alliance (http://www.mhealthalliance.org/) and The Center for Health Market Innovations (http://healthmarketinnovations.org/). The questionnaire was validated during a few initial interviews and the final version considered details of (i) the onset of a programme, (ii) its implementation and scale-up, including issues related to cost, financing, business model, human resources, infrastructure, technology and evaluation and (iii) the enablers and challenges in running it.

### Sampling

SJ sent email invitations to all 48 programmes in India listed at http://www.mhealthalliance.org/ at the time. Early interviews with those who responded led to other contacts. Only persons who had worked in organizations directly employing eHealth technologies were selected. Potential interviewees were invited to be part of the study that was outlined to them, and a positive response to the email invitation was taken as informed consent to participate. In all, we conducted 30 interviews, representing 28 organizations, with a few follow up clarificatory emails. Further attempts, over several weeks, to recruit interviewees did not succeed.

### Data collection

The interviews were conducted by SJ between March and September 2012, inclusive. Interviews were semi-structured around the validated questionnaire (Additional file 1). Notes were taken during each interview or immediately thereafter. In order to address the confidentiality concerns of the interviewees, the interviews were not recorded. The interviews were 25 to 90 minutes long, with an average of 55 minutes. Eight interviews were carried out face-to-face, 21 by phone and one by e-mail. In each case, complete annonymity and confidentiality were assured. Data collection was stopped when all those who had agreed had been interviewed.

### Data analysis

Descriptive analysis of the interview notes was done as follows: the replies of each interviewee were collated under the corresponding questions of the questionnaire and analyzed together in thematic clusters as in the questionnaire. Categorization of mHealth programs in India by the respondents turned out to be largely in accordance with that proposed by the mHealth Alliance [24] and this framework was adopted in this study. Other themes are presented as a direct compilation of the replies without an attempt to create a framework. Overall, an inductive analytical approach was adopted. This approach allowed us to reflect the rich diversity of experiences of the various mHealth stakeholders.

## Results

**Figure 1:**
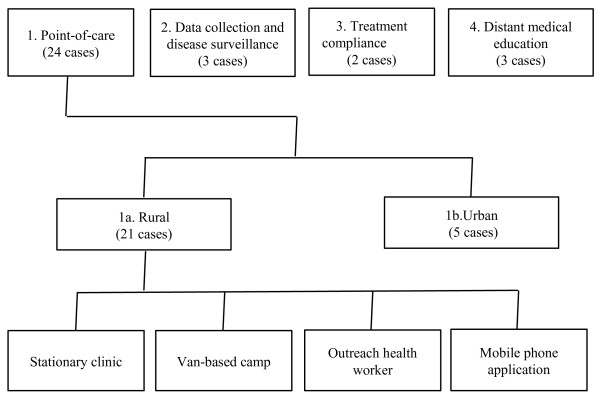
Categories of eHealth in India.

Largely in accordance with the framework used by the mHealth Alliance [24], the organizations interviewed were categorized as follows (Figure 1): (i) point-of-care support (24 cases), (ii) data collection and disease surveillance (three cases), (iii) treatment compliance (two cases) and (iv) distant medical education (three cases). These include five cases of (exclusive) developers of software or hardware that have been classified based on category of use. Some organizations’ activities fall into two categories and hence the number of cases exceeds 28. Point-of-care can be in rural (21 cases) or urban areas (five cases), with two technology providers figuring in both. We first consider the nature of work in each category before discussing their enablement by technology, the various challenges in running such programmes, and their finances. As mentioned above, the interviews were not recorded. Therefore, we do not include quotations in the Results. Although triangulation or other techniques of verifying responses were not used, interviewees from similar situations had broadly similar responses to a given question, confirming the reliability of the responses.

### Nature of work

#### Point-of-care

Telemedicine starts when doctors share their phone numbers with patients, in an informal way. This has been happening for decades in India. A subsequent effort involved medical equipment being installed in a car, and a specialist being consulted using the nearest landline. Current efforts are outlined below.

##### Rural and semi-urban programmes

The four main streams today are (a) stationary clinics, (b) van-based camps, (c) a technician working with a mobile phone and (d) mobile phone applications. Some organizations focus on one or two medical issues, such as diabetes and hypertension whereas others work across issues, and can span pre-natal care, malnutrition, dengue and HIV/AIDS, for instance.

(a) A stationary clinic (whether private or public sector) may already exist, which a non-governmental organization (NGO) or a city-based hospital equips with diagnostic hardware, a computer and an internet connection to enable tele-consultations with a specialist in the city. Illustratively, a general physician may not have the facility to record electrocardiograms (ECG), but will install the requisite machine and then make use of such tele-consultations. This care may or may not require video conferencing and the telemedicine aspect is sometimes hidden from patients. The primary care doctors are happy to be able to provide an additional category of care and popular areas for this modality include cardiology, neurology, nephrology and paediatrics. This connection of specialists to distant doctors may also be done internationally, and one Indian hospital has connections to 150 centres globally. The targeted beneficiary here is the primary care doctor who now has better facilities and access to the specialist, which in turn benefits the patient.

Another approach is to set up a new clinic. An NGO refurbishing an existing centre or setting up its own facilities may have specific communication strategies, including branding, to attract patients. The centre may be manned just by technicians, or by fresh medical graduates who will be connected to a specialist in the metro. There might also be a technician to help with the computer. The consultation may involve a simple telephone call to the doctor, or it may use the satellite connection provided for free by the public sector Indian Space Research Organization (ISRO). In addition to the consultation, patients may be provided free medicines and transport to and fro the consultation.

Training the manpower that will work in non-urban centres is important to the success of such a programme. In some cases, local people, including high school students, are recruited and trained at an urban hospital before returning to the community to administer primary care.

Another scenario in this category involves increasing the efficiency of a hospital’s specialists without reaching new patients. Several corporate hospitals routinely send their specialists to peripheral clinics, and telemedicine enables the doctors to provide care by analysing reports at the base hospital instead. In this situation there is only an exchange of patient records without any video conference. For one large private hospital this forms its only category of telemedicine.

(b) Care may also be provided by an outreach van that may be a stand-alone activity or complementary to a clinic (that may or may not be part of the same organization). In one example, a van carries nurses, general physicians and a technician, has a small pharmacy, and is equipped to take ultrasounds, ECGs and X-rays. Specialist tele-consultations take place in cardiology, neurology or critical care. In another case, there is specific screening for diabetes and diabetes-related complications like nephropathy and coronary artery disease. An eye hospital’s van programme may involve multiple distant doctors reviewing eye images in real time. The spectacles may be made in the van, or at the base hospital with subsequent distribution by a local NGO.

(c) Some programmes work through outreach health workers, especially the Accredited Social Health Activists (ASHAs) who are part of the Government of India’s (GoI) National Rural Health Mission (NRHM).

Largely, the programmes have been run in non-urban settings, although sometimes in urban slums, with those relating to mother and child health being the most popular. The ASHAs have been provided mobiles that are usually Java-based. The mobiles increase their stature in the community, and the message on the mobile is also taken more seriously than the spoken word. The mobile may have an algorithmic set of questions that the ASHA needs to ask the beneficiary, such as a pregnant woman or a patient. It also tracks whether she has made the requisite number of visits to the beneficiary and has spent enough time per visit. It improves the quality of care, including follow-up and emergency care, and the number of referrals. Some software specializes in case management. It can track the number of beneficiaries in particular programmes and will identify someone who has been lost to follow up. Monitoring the productivity of rural health workers, who normally work without supervision, is an important aspect of these programmes. A separate initiative involves instructional videos that are preloaded on a micro-SD card for a mobile phone, that is purchased in small shops. There are key messages on 14 health issues, such as hand washing, oral rehydration therapy and exclusive breast feeding, that are derived from a document that is endorsed by organizations of the United Nations. These serve to educate the ASHAs and beneficiaries directly as well. The expectation is that the programme will modify behaviour and reduce mortality, improve nutrition and so on. In some cases, the ASHAs receive modest cash incentives to bring patients to the centre from where there is a telemedicine consultation. Despite several challenges, as mentioned below, some of the ASHAs are highly motivated, contributing to the success of the programmes.

In a separate effort, more than 20,000 traditional birth attendants have been trained and given free home delivery kits. They also use diagnostic hardware that is tele-linked to doctors at a base hospital. Yet another kind of outreach consists of a technician who visits diabetic patients, for instance. Pictures of the eye, taken with a mobile phone, are sent to the hospital for an opinion.

(d) Finally, mobile phone applications can include health related games that can be played on low end mobiles, and sms-es for behaviour change or for reminders of visits to the doctor.

##### Web based care in urban areas

Aside from rural populations, some telemedicine is geared to urban users, who are targeted by for-profit companies. Some companies focus on primary and preventive care, creating awareness about health, diet and fitness, and working for behaviour change. One company has tied up with a private telecommunications company to send health tips to users’ mobiles. As part of this, proprietary content that is culturally more suitable for Indian users may be developed. They may also enable web-based consultations, with the patient reaching a nurse, a doctor or an ambulance from home. A suitable variety of doctors may be empanelled to handle multiple languages.

One sub-category of web-based care is that of follow-up telemedicine after surgery. Patients, primarily from tier 2 and 3 towns, receive a password to a website which enables communication with their doctor and an exchange of files after returning home. This category dominates the telemedicine practice of one hospital chain.

#### Data collection and disease surveillance

There are a large number of pilots concerned with data collection in eHealth, primarily mHealth. Relevant studies include the million deaths study (MDS) and medical certification of cause of death. MDS is the biggest electronic health-related data collection project in the world and is being done in partnership with GoI, which has provided access to its data collectors and the data already collected. Aside from collecting fresh data the participating organization is analyzing existing data and converting records to an electronic form. The results from MDS can sometimes differ widely from other GoI data, which should be useful inputs for better public health planning. A separate study concerns identifying long term risk factors for non-communicable diseases.

In terms of disease surveillance, one study, concerned with dengue, was a project to demonstrate that effective and up-to-date surveillance records can be created using mobiles instead of paper. A typical record consisted of the patient’s name (optional), gender, age group, location, disease, signs, symptoms and case-status (referred and treated). Also, a text entry field termed ‘notes’ was provided to record additional information. It typically took less than 10 seconds for a record to be created, and the submitted data was stored in a central database. The application also had a store-and-forward modality in case of a break in connectivity.

#### Treatment Compliance

Two programmes are concerned with treatment compliance, one involving patients and the other healthcare providers. The first concerns antiretroviral (ART) adherence, and works on the Mobile Technology for Community Health (MOTECH) platform, on a simple mobile. The patient selects options via an interactive voice response system and receives automated advice. The programme captures adherence to six specific regimens and the algorithms also identify common side effects. It is useful even in urban areas where patients may forget to ask questions during a doctor’s visit, may forget to take medicine, or may be lost to follow up if they don’t return to the clinic. This is a qualitative pilot study. It is a successor to an earlier pilot, for both urban and rural patients, where the preferred language, pitch of the voice and so on were assessed to make the system more usable and friendly.

The second programme involves managing, at scale, tuberculosis (TB) registrations and TB suspects. Through a web-based interface, the ‘directly observed treatment, short-course’ (DOTS) provider uploads data on a patient and the drugs taken. The system also tracks when the provider distributes a sputum collection container so that a map of TB suspects can be created. This programme works with GoI’s Revised National Tuberculosis Control Programme (RNTCP) to strengthen it, by using diagnostic and drug resources, also from RNTCP.

#### Distant medical education

Doctors’ education (either basic or continuing education) is a fairly active aspect of telemedicine in India today. Examples of such programmes are as follows. (i) One corporate hospital chain has conducted over 500 grand rounds through multi point video conferencing; (ii) A medical college has conducted 1500 workshops and conferences using ISRO’s connectivity or over the internet; and (iii) GoI has created the Pan African eNetwork for education and health, covering 42 countries, which is used both for clinical consultations and for continuing medical education. Thus, categories (ii) and (iii) are supported by GoI, but category (iii) is primarily delivered by private medical institutions.

### Technology

eHealth requires suitable technology, and interviewees reported on connectivity, software, hardware and analytics.

(a) *Connectivity*: The prime mover of telemedicine in India, ISRO, did proof of concept demonstrations in the 1990s. This was a pioneering effort, even globally. Subsequently, in 2001, it became a regular programme in certain government hospitals. However internet and mobile connectivity are much cheaper now. Moreover, satellite communication equipment is extremely bulky and cannot be taken to remote locations easily. So, although use of the ISRO satellite connection persists, it is decreasing. A special case of connectivity involves the (public sector) Indian Railways. Most of the tracks are hooked up to the landline telephone network, and one public sector initiative locates projects near railway lines for this reason.

(b) *Software*: For outreach workers using mobiles, data collection is not straightforward, given widespread illiteracy or semi-literacy. The questionnaires have to be primarily yes/no- or menu-driven. Data validation is important in terms of the length of the answer and the types of characters entered, and nonsensical responses should be rejected by the system. In one case, the application has been completely changed so that every question engages the user through audio and visual props. Although the software is sometimes designed to work on both Java and Android phones, it usually has to work on lower cost mobiles. One organization distributes mobile phones and also provides a field engineer who designs an application during the course of the project if required.

Software has also been created for electronic patient records, for processing, compressing and transferring images, for post-operative follow up and for back-end services. Commercial software tends to be compliant with Digital Imaging and Communications in Medicine (Dicom) standards and compatible with data formats from a wide range of devices. Some organizations use the open source MOTECH and may feed their improvements to the platform for other users.

(c) *Hardware*: Hardware usually records some or all of the following medical parameters: heart and lung sounds, blood pressure, temperature, blood glucose, skin and ear, nose and throat images, SpO2, ECG and pulse rate. The data is captured, stored and transmitted to a central server. Several vendors, or users, have developed their own device, with similar functionality. In one case it took six months to develop the technology, and a further six months to validate it in public sector hospitals. The company has obtained the IEC mark for its device and is now applying for the (European) CE mark. The device is usually sold, not rented.

(d) *Analytics*: In one case, the firm supplying the hardware subsequently does analytics on its clients’ data related to disease profiles, the kind of consultations per patient, drugs prescribed and diagnostic parameters of patients. It may also use economic and demographic data for modelling. This company does not rent or sell its hardware, and instead prefers to have a partnership with its clients.

### Challenges in eHealth

There are several challenges in this field of work, some pertaining to one or two programmes and others to several. These are listed below.

*Donors*: A major challenge concerns the tendency for donors to like the concept of mHealth and to require it of the NGOs they fund. The NGO hastily proceeds, without fully understanding the requirements on the ground. The programmes are therefore donor-driven rather than needs-driven. Donors may also prefer to give money for a specific disease, although patients require overall healthcare. Furthermore, donors (or clients or technology providers) sometimes prefer the latest technology, which can be a challenge to implement once users are comfortable with an earlier one.

*Government*: The GoI’s Planning Commission strongly recommends telemedicine. Nevertheless, there may be a lack of suitable policies, or lacunae in their implementation, that can prevent more effective telemedicine.

In a government hospital, for instance, neither is telemedicine mandatory, nor does a doctor earn more for this work. Therefore these doctors are often unmotivated to use this modality. In private hospitals, in contrast, a certain amount of van duty or other telemedicine may be made a mandatory part of the doctor’s job. In the largest government (satellite-enabled) programme which involves 425 set ups donated by ISRO, 85% are not in use due to bad management, insufficient incentives, or lack of technical staff or electricity. In this programme, although private hospitals constitute only 30% of the participating hospitals, they account for 70% of the tele-consultations, that are delivered on a non-profit basis.

Although there are examples of NGOs or private healthcare providers working with the government and doing a good job of creating telemedicine facilities and programmes, there can be several challenges in working with a government (at the state level or national). If a government agency is required to clear a programme or requires permission to be part of it, there can be large delays. More seriously, a change in the government may lead to a programme being axed. Also, by government guidelines, the number of people covered by a programme often serves as a surrogate for impact, which can be unsatisfactory. In terms of funding, there may be a good budget, but no assurance of how it will be spent. There needs to be a better matching of the needs and the financing, especially because government funding usually comes with the requirement that the money has to be spent within the year or be forfeited. Sometimes due to fear of misutilization much of the funds may remain unspent at the end of the year. Furthermore, government funding often targets secondary and tertiary care that requires hospitalization, instead of primary or preventive care, an exception being the ASHA programme.

In terms of health education, the multi-levelled administration can be inefficient in implementing complex programmes. Thus, a proposed 12-step process may be reduced to three steps by the time it is implemented, losing much of the meaning of the intervention. Yet another issue concerns corruption. By a minister’s own admission, preventive care projects are not lucrative, and therefore such projects tend not to be supported. Sometimes a state government issues a tender just for infrastructure, since this is where people can make illegal money, and does not plan for other elements of a programme.

*Telemedicine Society of India (TSI):* In 2003–5 TSI’s telemedicine hype lead to the creation of infrastructure and to ISRO donating bandwidth and equipment. Unfortunately, due to the inefficiencies of the public sector described above, the infrastructure has remained largely unused, leading to huge disillusionment in government.

*Records and data*: Many telemedicine projects are based on the electronic collection and storage of data. However the government either mandates a paper-based system or it fears that the people dealing with paper records will become redundant with a switch. Also, even if some data is collected electronically, the remainder has to be done by paper, lowering the incentive to shift to electronic records. Yet another challenge is that it is not trivial to collate data in a format in which it can be analyzed, and also map the data to the geographic area of coverage. Furthermore, as NGOs or governments use mobiles, they may suddenly have large amounts of data that they are not in a position to handle. They may then need to invest in human resources for data analysis.

*Technology*: There are several technological challenges in telemedicine. First, those who design the mobile applications may do so without much understanding of the ground reality, making them sub-optimal. Second, as mentioned above, there is a trend to constantly upgrade software while the existing one is still functional. Instead of implementing a project with the current software, money is spent on developing and piloting a new version. Low end mobiles are widespread, but cheap smartphones such as the open-source Android devices would be better suited for people with poor literacy, and for applications that employ a global positioning system (GPS) tracking function. However if one upgrades to a smartphone, one needs an application that may no longer run on a simpler phone. Thus there may be rapid obsolescence of the currently used hardware and software. Third, the novelty of the technology has required extended learning and optimization periods for both manufacturers and implementers, thereby slowing or decreasing its spread, especially in the early years. Fourth, the electric supply and network coverage can be poor, which is sometimes circumvented by the store-and-forward modality. If the government subsidised it, a satellite connection could be used where internet connectivity is absent, but no new satellite-based connections are being established. Fifth, companies that were pioneers in this area were forced to come up with their own technical solutions. Thus, hospitals may use proprietary software that is not Dicom compliant, and therefore not transferable across hospitals with different patient flows, employment structures and so on. As and when modalities for scale up across organizations are worked out, standardized software will become essential. Thus, there are several technical challenges, and yet human resources are a far bigger issue, as discussed further below.

*Fragmentation of programmes:* The most successful eHealth programmes are driven by organizations outside the government, whether for-profit or non-profit. However even if a programme covers most of a state, it will be offered by a single organization, possibly in partnership with the government. An exception is an ophthalmology programme, developed by a private hospital in one state, that the government of another state is copying. It is being rolled out as a public-private partnership, with the original hospital acting as a consultant to the private partner in the latter programme.

Thus, an over-arching issue concerning telemedicine, whether amongst those who run programmes, or those who develop the software or hardware, is that each initiative is running on its own, without the sharing of experiences. Also, there isn’t much research in this area. This wastes resources due to the duplication of effort, and also impedes scale up.

*Communication*: There are different kinds of challenges related to communication. First, since IT and medical professionals don’t really understand each other, developing appropriate software is not straightforward. Second, for the educational projects, it is important for beneficiaries to be engaged so that there is acceptance of the information. Not everyone believes the messages concerning washing hands and sleeping properly for instance. Thus, health has to be made an interesting topic if such educational programmes are to succeed.

*Logistics*: There are logistical challenges, especially when working in remote areas. (i) It can take three months to plan a van trip. The planning includes identifying NGOs and donors, educating patients about which conditions need medical attention and motivating them to attend the camp. The follow-up of patients is also non-trivial. A specific challenge is that the van returns to the city every evening. It would take separate incentives to get doctors to stay on the road overnight to enable them to reach the most distant patients. (ii) Patients may come to the health centre only in the morning, although the specialist is available for tele-consultation only in the late afternoons. (iii) After the tele-consultation, there can be the challenge of patients’ access to drugs. To deliver drugs to a rural area, one organization tied up with public buses, and logistics were worked out according to the bus schedules. Although the same organization also tried to work with unbranded generics, they found that the better distribution channels of branded generics was worth the extra cost.

*Manpower*: The shortage of skills is evident in a variety of settings, as follows. (i) Although supposedly educated up to middle school, some ASHAs are illiterate and can barely answer a call. Therefore learning how to use the phone can be a challenge. Their professional training may also be poor, leading to low quality counselling of beneficiaries. (ii) Technicians who work in the telemedicine centres are not skilled in computer use and need to be trained. This can lead to high training expenditure and staff shortages. (iii) A specific challenge in MDS is the narrative part of the autopsy where health workers need to make accurate notes based on what family members say. This requires a special skill. (iv) The NGOs and research groups in health and population studies, and in the government, may find technology challenging. There may be reluctance to learn something new, and even if provided a ready product they will not necessarily make full use of it. (v) A doctor needs special skill to interact with rural patients, being empathetic and humble, and discussing other things before health issues. A change in doctor can lead to a big drop off in the attendance at a rural facility if the new person lacks these skills. (vi) The doctor must also be flexible, and be able to function with the few facilities available in a rural setting. (vii) People shifting to other jobs is another persistent challenge. After some experience, a fresh medical officer will soon get a higher paying job elsewhere. Women birth attendants may get married, or pregnant, and leave. To tackle this issue, one organization only employs retired doctors, who move less often. (viii) Some of the challenges relate to working with technicians at a distance. Illustratively, one ophthalmology hospital does not allow a technician to dilate the pupil since he would be unable to deal with any side effects, and this limits the care that can be offered. (ix) Even staff in rural areas who have been trained may need re-training, sometimes weekly.

*Consulting the independent specialist:* A challenge specific to tele-consultations relates to the delay and hassle involved in operating the computer and other technology without assistance, waiting for the connection and so on. Further, billing and transferring money electronically means that doctors have to pay taxes on this income. Unless there are 30–50 consultations a day it is usually not worth the specialist’s time, and this may not have happened yet.

*Attitude*: Aside from skills, the attitude of everyone involved in telemedicine plays a very important role in its success. (i) Implementers need to have contagious enthusiasm for their projects. If they take the health workers into confidence at every stage of the project, the latter develop a much needed sense of ownership of the project. (ii) An ASHA is often more preoccupied with her own household than with her official work. (iii) A common problem is that a rural doctor may be too ‘proud’ to consult an urban specialist, despite patients’ requests. (iv) Users’ acceptance of both technology and mode of healthcare delivery is important, and convincing them may take considerable effort. (v) Services related to family planning, contraception and abortion cannot be implemented easily in South India, which is more conservative. Also there are more qualified allopathic medical practitioners available in the south, so a project cannot be run with the less qualified Registered Medical Practitioners who will be accepted in the north.

#### Policies and regulation

A number of government policies can affect mHealth operations, yet different categories of programs will be affected by different policies. For example, those aimed at improving rural infrastructure such as electrification and mobile network coverage are likely to have a positive impact on most programs. This is happening in certain regions of the country, although other regions are still neglected. Policies that make mHealth duty compulsory for doctors working in public hospitals would benefit telemedicine programs that involve collaboration with such hospitals. A policy that promotes and supports the funding of the complete substitution of paper records with electronic ones will contribute to the expansion of data collection and disease surveillance programs. Such policies are much awaited by the eHealth community.

Regulation of mHealth in India is an emerging issue and whereas the telemedicine community came up with guidelines in 2003, these apparently had many wrong premises, such as the assumption that a doctor is present to sign a patient’s consent form, and this has lead to criticism. Currently there are no national guidelines for mHealth practice, which gives the implementers much freedom. Illustratively, one private tele-opthalmology operator intentionally does not inform patients about the fact that the pictures of their retina are transmitted to a remote location where they are analysed by a specialist doctor. In general, most telemedicine operators reported that they comply with regulations of standard medical practice laid down by the Medical Council of India and state regulatory bodies. For example, an operator of mobile telemedicine centres manned exclusively by non-medical staff could not deploy a mobile pharmacy because only qualified pharmacists are allowed to dispense prescription drugs. Since no pharmacies were present in the concerned rural areas, drugs were dispensed in a city and shipped to villages on scheduled buses as described in the section *Logistics* above.

### Finances and sustainability

One of the biggest challenges facing most of the programmes concerns funding and sustainability. The sources of funds (or other crucial support) for each programme are listed in Additional file 2. These are primarily philanthropic donors, government (that usually provides non-cash support), cross-subsidy or revenue, and rare cases of venture capital. The financial sustainability of each programme is also discussed. In terms of government-run or supported schemes (including those involving ASHAs), these will be sustainable so long as the government continues its support. In several cases, sustainability is dependent on patient volume. Thus there are geographic limitations on where such programmes can run. Low-cost efforts such as health information, where corporates see value in large-scale visibility, are sustainable due to the latters’ support. For-profit endeavours that depend on venture capital investment are yet to prove their sustainability, even in urban areas.

The costs for some aspects of the programmes are listed in Additional file 3. These include capital expenditure such as that for a van (Rs. 25–30,00,000), a stationary clinic (Rs. 1–2,00,000 for a basic clinic or Rs. 5–6,00,000 for an eye clinic) or a mobile phone (Rs. 4,500 for a mid-range model), and running costs such as doctors’ salaries (Rs. 6000 for a fresh graduate in a rural area), incentives to ASHA workers (Rs. 100 a month to bring patients to a tele-medicine centre) or mobile phone connection charges (Rs. 199 for a month’s rental for biosurveillance work). Since most of the programmes are run by non-proft organizations and are geared to poor patients, running costs tend to be a small fraction of what would be charged by a for-profit entity in an urban environment, for example.

A specific mention should be made of how doctors were paid for their involvement in eHealth. In the most successful programmes, run by large non profit hospitals, doctors were not paid beyond their regular salaries. In government hospitals, too, there was no separate fee to a doctor. In the less successful website-based consultation programs doctors were paid via bank transfers by the website.

## Discussion

Every sixth person in the world lives in India. The availability and affordability of good healthcare is highly variable for different segments of the population, and therefore understanding the status of a given health system in the country is important to both national and global health. In contrast to previous articles on eHealth in India, which often focussed on single programmes, this study reviews the current scope of work in this field from the points of view of designers, implementers, evaluators and technology partners of a variety of programmes. However we have not quantified our data, or done a stakeholder analysis, which is left for a separate study.

The programmes of this study fall into four of the five eHealth themes previously identified in the literature [24]. The fifth, emergency medical response, could not be included due to our inability to recruit relevant interviewees. Also, we did not encounter programmes for handling poor drug inventory and supply chain management [25] or for streamlining financial transactions or mitigating fraud and abuse in drug authenticity or patient identity for instance [11] as reported from other developing countries. eHealth in India has not focussed on high end technologies such as robotic surgeries. These make interesting news but are not particularly relevant to efforts at changing health outcomes for a large population. Indeed, the programmes that fared well employed low cost and simple technologies. Whereas the literature reports challenges due to the high cost or complexity of technology [24], we did not identify any such case in India although (i) the bulkiness and complexity of satellite connectivity may lead to its obsolescence soon and (ii) doctors may find even operating a computer an irritation when there are insufficient numbers of patients requiring this modality. Interestingly, the low cost hardware was often developed indigenously. Biomedical innovation is a recent but documented phenomenon in India [26] and this study contributes to such literature. Suitable software is also developed locally, as happens in Africa as well [27].

The eHealth programmes face a range of challenges, several described elsewhere [11,24,27]. However some stand out for their likely persistence. The shortage of health care professionals, especially in rural areas, seriously impacts eHealth operations. Organizations struggle to train and retain skilled human resources. There is a scarcity of published research on the types of incentives that are effective in promoting the adoption and proper use of mHealth by such health professionals [24] and our study provides novel insights into this issue. In the rural setting, the retention of health workers is enabled chiefly by (i) training local residents who return to their village on completion of training and (ii) explaining to health workers the benefits of using mobile technologies, such as the increase in their efficiency and enhancement of their social status. In the urban setting, non-hospital based tele-medicine centres should select doctors based on their (a) ability to communicate with village folk, (b) ability to manage the distant patient who has limited resources, (c) preference in working in such an office rather than a clinic and (d) willingness to stay in a given organization for a few years rather than moving on soon. The largest cities are a preferable location for such a telemedicine centre due to the oversupply of doctors there. Overall, the private sector (non-profit or for-profit) has been more effective in ensuring that where doctors are supposed to do telemedicine in addition to regular hospital duties, they do so. The lack of suitable incentives remains a major problem in the public sector (as noted in the need for providing higher quality of regular healthcare also [7]), and amongst doctors with their own practice. Interestingly, whereas research in other developing countries has suggested that medical professionals, including doctors, find mobiles valuable for their own education [24], our study confirmed this phenomenon with rural workers but not urban doctors.

mHealth programmes that collect data at unprecedented speed are also affected by a shortage of skilled manpower. We have found that neither the relevant government programmes nor private organizations may have the human resources capable of analysing and applying the findings in a meaningful way. Because such programmes often have public health importance, their future will depend both on close involvement with relevant government agencies and on new policies that promote a complete substitution of paper-based systems with electronic ones. Implementing this on a large scale will be a non-trivial task.

Likewise, the scale up of successful treatment compliance pilots can only happen in close coordination with public sector programmes that provide free diagnosis and treatment to significant fractions of patients with HIV/AIDS or TB [28,29]. As suggested by previous research [24], partnering with existing public health programmes is a preferable general strategy for the scale up of mHealth pilots, but with a few exceptions this is yet to happen in India.

The eHealth implementers of this study, whether for-profit or non-profit, have been supported by, or have engaged with, government institutions in a range of ways although the latter can sometimes be a challenge: (i) accessing satellite connectivity for free; (ii) collaborating with the ASHA programme, the Registrar General of India (for the MDS) or the RNTCP; (iii) collaborating with government hospitals for screening newborns, a programme in which the state government pays the salaries of the relevant personnel, who have been selected by the private hospital; (iv) arranging van-based camps after discussion with the district collector who indicates where they should be, since this was more effective than approaching the head of a village directly and (v) occasionally using the government’s rural health centres for tele-medicine consultations. Most government facilitation is through its infrastructure or personnel, not through funding. Notably, such private-public collaborations were more successful than entirely public programmes. In the ISRO-promoted telemedicine initiative and the government-managed medical education programme, most sessions have been delivered by private organizations on a non-profit basis, and these programmes continue to grow. Nevertheless, there are no rigorous evaluations of the effectiveness of this educational modality [13].

This issue of the cost-effectiveness of eHealth is a matter of debate globally, and rigorous research in this area is missing [13,30]. Whereas none of the interviewed organizations could provide us with such data, there was a general belief that, given poor health awareness and the scale of neglect of health care in rural India, any initiative that improves the situation is justified. Therefore several organizations have experimented with eHealth delivery and sustainability models over the past decade, motivated by a desire to bring their services to rural areas rather than by hard evidence of the cost-effectiveness of such efforts.

As elsewhere [25] our study included several unsustainable pilots. The programmes that appear to be financially sustainable are: (a) government programmes; (b) private initiatives supported by the government, including those run in remote areas or those run over a large area such as an entire state; (c) programmes run by large non-profit hospitals; and (d) in one case corporate sponsorship was enough to sustain the low cost activity of dissemination of health information. With regard to category (c), there are pricing/financing models where richer patients in the city-based hospitals subsidize the expenses of treating poorer urban or rural patients. The larger hospitals also have enough doctors for the telemedicine programme. In cases where the running expenditure is met by patient fees this will work even though the rates charged can be an order of magnitude less than what is charged in urban areas so long as there is a sufficient number of patients. Patient turnout depends on the quality of the staff, but also on the location, since rural or remote areas may have a low population density.

Overall, we believe that the strengths of this study are that it has interviewed a very diverse set of organizations, that heavily overlap known categories from other studies in the developing world, and are representative of the field of eHealth in India today. We have been able to catalogue the nature of work, the challenges and the finances that affect these areas of work. There are, however, some weaknesses. We were unable to record the interviews, and thus if there were nuances that would have been picked up in the subsequent analysis of recorded interviews, we may have missed them. Although we interviewed a wide set of organizations, the number for most of the categories is low. Point-of-care (24 cases), and within that rural care (21 cases), is the only set of activities involving interviews in the double digits. We believe that these weaknesses notwithstanding, the analysis done would be useful to policy makers and to entrepreneurs or others wishing to enter this field.

## Conclusions

eHealth in India is primarily targeted at those unreached by modern medicine, and it is a challenge to run for-profit programmes where most beneficiaries are poor. In terms of scale, charitable organizations, particularly those that do not generate their own revenue, are usually unable to impact millions of beneficiaries. One of their biggest challenges is finance. Since the fees are already very low, it may not be possible to try and increase the number of beneficiaries by further reduction. There is also a danger in raising the fees, since this may make the service beyond the reach of current beneficiaries. Thus, it may require other efforts to put the programmes on a stronger financial footing. Aside from steps already mentioned, such as improved infrastructure, compulsory telemedicine duty for doctors in government hospitals, and replacement of paper records with e-records for data collection and surveillance, the government could consider the following steps:

1. Capital support based on track record. Over time, an eHealth activity could receive incrementally increased support, based on annual performance. Half or more of the capital expenditure could be borne by the government.

2. Provide matching funds per patient. The government could consider matching the doctors’ fees charged to patients (and perhaps the cost of medicines or diagnostic tests), for non-profit eHealth providers in particular.

3. Support training programmes. Since training is a major expense, this could be supported just as other skill-building or educational activities in the country are supported by the government. Awareness programmes, including health-based games, could conceivably also be given matching grants, especially when offered by non-profit organizations.

4. Tax benefits to any hospital offering a certain number of free teleconsultations per year.

Separately, and as recommended by the authors elsewhere [26], the government should provide greater clarity on the specifications that novel mHealth devices must meet, to help entrepreneurs avoid the cost and delay of obtaining international certifications. Likewise, greater public sector procurement would help entrepreneurs in this sector.

Some of the policy recommendations above are based on tracking patient use. For e-health initiatives, by definition, record keeping should not prove a challenge.

In the absence of such interventions, eHealth will essentially be left to the government if it is to have a large impact. Our study indicates, however, that programmes left solely to the government are not very effective. A partnership between the government and either a for-profit or a non-profit is the most likely to succeed. Even so, the lack of suitable human resources in rural areas will be a challenge to any large scale effort.

## Abbreviations

ART: Anti-retroviral treatment; ASHA: Accredited Social Health Activist; CSR: Corporate social responsibility; DICOM: Digital Imaging and Communications in Medicine; DOTS: *Directly observed treatment, short*-course; ECG: Electrocardiogram device; GoI: Government of India; GPS: Global positioning system; HIV/AIDS: Human immunodeficiency virus/acquired immunodeficiency syndrome; ICT: Information and communication technologies; ISRO: Indian Space Research Organization; MDS: Million deaths study; MOTECH: Mobile Technology for Community Health; NGO: Non-governmental organization; NRHM: National Rural Health Mission; RNTCP: Revised National Tuberculosis Control Programme; sms: Short message service; TB: Tuberculosis; TSI: Telemedicine Society of India

## Competing interests

GS’s research is funded by Institut Merieux (IM), Lyon, which has financial interests in the medical technology industry. However IM does not have any interest in telemedicine per se. Its sole role was in general funding to research in GS’s group, as indicated below. It did not play any specific role in the study such as proposing it, designing it, or analyzing the results.

## Authors’ contributions

GS proposed the study. SJ performed the interviews. SJ and GS analyzed the interview notes and wrote the manuscript. Both authors read and approved the final manuscript.

## Pre-publication history

The pre-publication history for this paper can be accessed here:

http://www.biomedcentral.com/1472-6947/14/1/prepub

## Supplementary Material

Additional file 1 **The questionnaire used as a template for the interviews.** The questions were modified suitably if the interviewee was active in other aspects of eHealth. Also, only relevant questions were asked of an interviewee. Click here for file

Additional file 2 For 21 organizations, the nature of the organization, the categories of work undertaken, its sources of funding and comments about its financial stability. Click here for file

Additional file 3 The costs of some elements of eHealth in India today. Click here for file
