# Supplementary material for: In eHealth in India today, the nature of work, the challenges and the finances: an interview-based study
Source: BMC Med Inform Decis Mak. 2014 Jan 6;14:1. doi: 10.1186/1472-6947-14-1 (PMC3893581; doi:10.1186/1472-6947-14-1)
Supplement: Additional file 1 — The questionnaire used as a template for the interviews. The questions were modified suitably if the interviewee was active in other aspects of eHealth. Also, only relevant questions were asked of an interviewee. [file 1472-6947-14-1-S1.pdf]

**mHealth questionnaire**

About your venture/program:

1. What programs are you doing in mHealth/telemedicine? Since how long? Who is funding the program (donors, government, end users, patients). What mobile/tablet?
2. Are these pilots or scaled up programs?
3. Do you focus on a single health issues or many? Why?
4. Was the program integrated into existing healthcare structures in the region or new healthcare facilities and medical stuff were employed? How/did you identify real needs and demands of target beneficiaries, local health priorities (also cultural/gender)? Was your program initiated upon suggestion of the community health centres/government/funds donor/technology provider? What was the rationale to employ mHealth?
5. How did you achieve program uptake by end-users (promotion, education)?
6. How much coverage in what time (number of patients, end-users)?
7. Do you collect data on the effectiveness of the program in improving patient outcomes? What monitoring and evaluation metrics do you have for your program?
8. Was there any technical challenge (software and hardware) around which you had to innovate or partner with someone (possibly abroad)? Does your device operate in off-line mode?
9. Did you have to change/alter the profile of your program when faced with a failure?
10. What was the business model (if any) of the programs?
11. How do you want to achieve program sustainability (funding, government, community)?
12. Overall, what has been surprisingly easy and what were the major challenges?

About the general situation in India:

13. Why have so many pilots in India not been scaled up?
14. According to a recent WHO report, only 7 percent of mHealth initiatives in developing countries have been evaluated in randomised field trials to show efficacy versus “placebo” intervention. Why so few?
15. What are the drivers of growth on mHealth currently and what are the impediments to growth (financing, infrastructure or software, literacy, mindset of patients/doctors)?

16. Is satellite connectivity giving way to GPRS/mobile connection nowadays? What is the network coverage (% of population)?
17. Who else in India is doing this kind of work?
18. Which type of mHealth/telemedicine programs are the most successful in India? Why do you think they succeeded?
19. What are the categories of mHealth, in India?
