# Supplementary material for: In eHealth in India today, the nature of work, the challenges and the finances: an interview-based study
Source: BMC Med Inform Decis Mak. 2014 Jan 6;14:1. doi: 10.1186/1472-6947-14-1 (PMC3893581; doi:10.1186/1472-6947-14-1)
Supplement: Additional file 2 — For 21 organizations, the nature of the organization, the categories of work undertaken, its sources of funding and comments about its financial stability. [file 1472-6947-14-1-S2.pdf]

**Additional File 2.**

| | Case | Type of work[$] | Beneficiary* | Source of funding | | | | | Financial stability of the programme, and related comments. |
|---|---|---|---|---|---|---|---|---|---|
| | | | | Donor | Government | Cross-subsidy | Revenue[#] | Venture capital | |
| 1. | Private hospital and its associated non-profit foundation | Cooperation of S and V | N | | Yes (existing hospitals) | Yes | Yes | | Financially stable. A successful scale up in private-public partnership with a state government. The government is contemplating taking this programme all-India. |
| 2. | Government hospital | S, E | Both | | Yes | | | | Stable, dependent on government funding |
| 3. | Non-profit | S, I, V | N | Yes | Yes (ASHA programme) | | Yes (experimental) | | Dependent on donor funding. Planning expansion to one other state. Cannot go further due to lack of funds. |
| 4. | Non-profit hospital | S, V | N | | | Yes (at start up) | Yes | | Self-financing if good patient volume. Another state has copied this model. |
| 5. | Non-profit hospital | S | N | Yes | Yes (doctors in government primary health centres) | Yes | Yes | | Self-financing if good patient volume. |
| | | V | N | Yes | Yes (satellite) | Yes | | | |
| 6. | For-profit hospital | E, V | N | Yes | Yes (satellite) | Yes | | | The work cannot be scaled up without funding. |
| 7. | For-profit hospital | S, E | Both | | Yes (satellite + others) | Yes | | | Sustainable only as a CSR activity. |
| 8. | Non-profit hospital | V | N | Yes | Yes | Yes | Yes | | Constant struggle for donations |

| | | | | | | | | | |
|---|---|---|---|---|---|---|---|---|---|
| | | | | | (satellite) | | | | generates uncertainty about the programme's future. |
| 9. | For-profit hospital | S | Near U | | | | Yes | Yes | Business model is still evolving. |
| 10. | For-profit company | S | U | | | | Yes | Yes | Business model is still evolving. |
| 11. | International non-profit | E, D | N | Yes | Yes (ASHA programme) | | | | It rides on the ASHA programme. It is not interested or capable of enlarging scale on its own. |
| 12. | International non-profit | S | N | Yes | | | Yes | | |
| | | S, D | N | Yes | Yes | | | | TB follow up programme rides on a government programme (a belief that for scale up,  the government should fund and the private sector should deliver) |
| 13. | Not-profit foundation linked to for-profit hospital | S, E | Near U | | Yes (satellite) | Yes | Yes | | Only sustainable as a CSR programme of the hospital. |
| 14. | Non-profit hospital | S, V | N | Yes | Yes (satellite) | Yes | Yes | | Cannot expand without further funding. |
| 15. | For-profit company | I | U | | | | Yes | Yes | Business model is still evolving. |
| 16. | Non-profit research organization | I | Both | Yes | Yes (existing programme of one state government for one programme) | | | | Business model is still evolving. |
| 17. | Non-profit hospital | D | Both | Yes | Yes (mainly | | | | Very large scale due to GoI |

| | | | | | | | | | |
|---|---|---|---|---|---|---|---|---|---|
| | and foreign non-profit | | | | existing infrastructure ) | | | | involvement. |
| 18. | Non-profit organization | I | N | Yes | Yes (ASHA programme) | | Yes | | Business model is still evolving. |
| 19. | Non-profit academic institute | D | N | Yes | Yes (existing infrastructure ) | | | | A pilot study not intended to be scaled up. A belief that the government would have to support the programme if it had to be scaled up. |
| 20. | Non-profit organization | I | Both | Yes | Use of logo | | Yes (corporat e sponsors hip + user charges) | | Corporate sponsorship enabled large scale up. |
| 21. | Non-profit company | D | Mainly N | Yes | Yes (ASHA programme) | | | | The programme cannot be scaled up without government funding. |
| 22. | International non-profit organization | D | U | Yes | | | | | The programme cannot be scaled up without government funding. |

*Beneficiary could be Non-urban (N), Urban (U) or both.
# Revenue could be from patients or other sources such as advertising.

$ Abbreviations for the categories of work
S Stationary telemedicine
V Telemedicine van
E Education of health professionals or activists
I IT-enabled health information for patients
D Data collection or monitoring
