# Supplementary material for: In eHealth in India today, the nature of work, the challenges and the finances: an interview-based study
Source: BMC Med Inform Decis Mak. 2014 Jan 6;14:1. doi: 10.1186/1472-6947-14-1 (PMC3893581; doi:10.1186/1472-6947-14-1)
Supplement: Additional file 3 — The costs of some elements of eHealth in India today. [file 1472-6947-14-1-S3.pdf]

Additional File 3.

The costs of some elements of eHealth in India are mentioned below:

1. The costs for a programme that is based on the ASHAs' participation: A mid-range phone costs Rs. 4,500/- and the running cost is Rs. 50 per month for the sms-es sent to each ASHA. She is not paid extra for using a mobile, although she may be paid to attend training. In one programme each ASHA is paid Rs. 100/- per month to bring patients to the telemedicine centre. Another organization has found that there is need for one supervisor per 50 ASHAs. Also, technology is only 33% of the entire programme cost, with the cost of training being more than that.

2. A fresh graduate medical officer is paid Rs. 6,000 a month at a rural centre, but with a little experience will soon be offered Rs. 10,000 elsewhere, and leave.

3. A rural shopkeeper connects patients to an NGO's telemedicine centre using his/her mobile, or through the internet, and makes a small profit. The NGO charges a fixed rate and allows market forces to determine the provider's charges to customers. Illustratively, the NGO charges the shopkeeper Rs. 10 per phone consultation and Rs. 30 or Rs. 70 for a randomly-assigned or preferred doctor, respectively, in an internet-based consultation. However a patient who is a 'Below Poverty Line' card holder would pay less than others. The Rs. 70/- per consultation is a price that approaches break-even for the NGO.

4. It costs Rs. 1–2,00,000 to set up a basic medical centre in a semi-urban or rural area. In such a centre, 20–30 patients a day are sufficient to break even. (b) A more advanced centre, that includes diagnostic lab facilities, costs much more, and requires many more patients to break even. In the latter situation the main revenue is from diagnostic tests. Also, selling medicines can generate more profit than the consultation fee.

5. Eye care, sustained by the revenue from drugs, glasses and lab tests, is a special case in terms of the business model. It costs Rs. 5–6,00,000 to set up a vision centre that has basic ophthalmology equipment, a computer with suitable software, spectacles, drugs and a conventional digital camera adapted in-house to take pictures of the eye. Blood tests may also be performed. The drugs, glasses and lab tests are priced much cheaper than in the city, but the centre can break even provided there are enough patients.

6. In one non-proft eye-care programme, a doctor in a city-based hospital, is connected to six vision centres in smaller towns, each of which is manned by two technicians. Each centre serves a population of 50-60,000. Each patient pays Rs. 20 for three visits or for three months, whichever happens first, and the patients pay separately for drugs and spectacles.

7. A for-profit eye-care organization is rolling out a screening and treatment programme in district towns. It has set up a hospital outside a major metro, where a junior and senior opthamologist have a fixed salary of around Rs. 70,000 and Rs. 1,20,000 per month respectively.

8. The capital expenditure for a van is Rs. 25–30,00,000.

9. In the biosurveillance work using a mobile phone, a Rs. 199 monthly rental package (with two Gigabytes of data transmission capacity, limited number of free sms-es, and free voice calls

within a closed user group) was more than adequate for each health worker.

10. For one technology provider, the costs were the same for the development of the software and hardware on the one hand and on their validation and certification on the other. The cost of certification was high because it was by western agencies.

11. It costs Rs. 30 per month to receive one health tip per day on a mobile phone.

12. The 2GB card that is loaded on a mobile phone costs Rs. 80. This should go down with larger orders.

13. The health games are priced at Rs. 2–5 each. This is 10-fold below the market price but it has allowed break even. Corporate sponsorship eventually allowed one million downloads.
